# Untapped potential: graduate students as catalysts in scientific discovery

**DOI:** 10.3389/fpsyg.2026.1746739

**Published:** 2026-04-22

**Authors:** Hannah B. Love, Ellen R. Fisher, Bethany K. Laursen, Ellyn M. Dickmann

**Affiliations:** 1 Divergent Science LLC, Fort Collins, CO, United States; 2 The University of New Mexico, Albuquerque, NM, United States; 3 Michigan Institute for Clinical and Health Research, University of Michigan, Ann Arbor, MI, United States; 4 Dickmann and Associates LLC, Loveland, CO, United States

**Keywords:** Archintor™, graduate student, network analysis, research teams, science of team science, scientific teams, social network analysis, team science

## Abstract

There is no universal blueprint for assembling scientific teams or co-creating knowledge. Yet, one critical and often minimized component in team research is the role of graduate students. Although funding agencies increasingly require the inclusion of graduate students as project personnel, little is done post-award to evaluate or support their integration into a team’s knowledge network. This neglect risks reducing graduate students to a transactional means of accomplishing project objectives rather than ensuring their genuine inclusion as knowledge contributors. This perspective article argues that graduate students are a missing or overlooked catalyst in scientific discovery teams. Using illustrative field cases and social network diagrams, we demonstrate the transformative impact of meaningfully integrating students and the high cost of their exclusion. Our preliminary observations suggest that graduate students can and do generate new knowledge, fulfill a multitude of roles on teams, and shape collaborative practices in distinctive ways through their influence in the network and the diverse perspectives and backgrounds that they bring to research teams. The article concludes with a roadmap to promote future research.

## Introduction

Imagine a meeting between interdisciplinary principal investigators (PIs), hashing out the next steps in a complex research project. After intense discussion, one PI concludes, “We will have my graduate student do that.” The student was not present, did not contribute to the decision-making, and likely lacks critical context for success. Yet, they are now tasked with advancing a central component of the project. This scenario highlights just one problematic pattern wherein students are charged with carrying out research ideas they were not invited to co-develop or co-create. Of broader concern, we have observed that graduate students are often a missing or overlooked catalyst in scientific discovery teams, which should open lines of crucial inquiry and action.

In the above scenario, the PIs, from different scientific disciplines, integrated their disciplinary expertise to develop a pathway toward solving a complex scientific problem. In the literature, this is known as breakthrough science (Cravens et al., 2022), convergence research (Peek et al., 2020) or knowledge integration (National Academies of Sciences Engineering and Medicine, 2025). How would solving this scientific problem be transformed if the graduate student was instrumental in both shaping, executing, and implementing the solution, while being actively engaged in an inclusive team network? Our preliminary observations suggest that graduate students can and do generate new knowledge, fulfill a multitude of roles on teams, and shape collaborative practices in distinctive ways through their influence in the network and the diverse perspectives and backgrounds that they bring to research teams.

## Existing literature

Solving complex problems requires effective disciplinary depth and interdisciplinary breadth (Bosque-Pérez et al., 2016; Eigenbrode et al., 2007; National Academies of Sciences Engineering and Medicine, 2025). Graduate students often bring bold ideas, awareness of the recent literature, growing scientific knowledge, and determination. These assets can contribute to the creativity, collective learning, innovation, and problem-solving on research teams. Graduate students’ input, however, may be ignored because they often lack positional power and certifications, and they may be perceived as being in the early stages of their scientific training. Preliminary observations and literature indicate that another way is possible. As catalysts of innovation and integration, graduate students can be connectors in the network, knowledge brokers, and leaders who help create conditions where others’ ideas and collaborations can flourish (Hoffmann et al., 2024; Koseoglu et al., 2022). This brief literature review highlights gaps in the literature and opportunities for future research.

### Training for graduate students

Studies focusing on graduate students’ roles in research teams are limited. Existing work emphasizes training to work on interdisciplinary teams of other graduate students (Borrego and Newswander, 2010; Graybill et al., 2006; Knowlton et al., 2014; Morse et al., 2007; Ryan et al., 2012). In addition, there are articles on undergraduate teams (Khuri, 2015; Love et al., 2022; Misra et al., 2009; Tripp and Shortlidge, 2019; Werth et al., 2022) and a handful of studies about team science training for graduate and undergraduate students together (Misra et al., 2009; Vogel et al., 2012).

### Team roles

Likewise, little research documents the roles graduate students can fulfill on science teams. We illustrate here how three existing frameworks of team roles might be developed to accommodate the unique contributions of graduate students.

#### Roles of graduate students: Belbin’s team roles

Belbin’s Team Roles model describes nine behavioral styles divided into three major categories: Thinking (Plant, Monitor, Evaluator, Specialist), Action/Task (Shaper, Implementer, Completer Finisher), and People/Social (Coordinator, Teamworker, Resource Investigator) (Belbin, 2010). Graduate students can contribute to team effectiveness across all three of Belbin’s role groupings: they enact thinking-oriented roles by generating ideas, evaluating approaches, and providing specialized expertise; people/social-oriented roles by facilitating collaboration, mentoring peers, and supporting productive team dynamics; and action/task-oriented roles by implementing methods, coordinating tasks, and ensuring progress toward project completion (Aritzeta et al., 2007).

#### Roles of graduate students: Malcolm Gladwell

In his book *The Tipping Point*, Gladwell describes three social roles driving transformative change: Connector, Maven, and Salesman/person (referring to roles). As this relates to graduate students, a Connector is someone who collaborates across labs, connects faculty and students, and bridges disciplines. A Maven graduate student accumulates knowledge, engages in learning, and is a trusted source of information. Finally, the Salesperson is a graduate student who can motivate others, communicate science clearly, and align collaborators (Gladwell, 2000).

#### Roles of graduate students: Stephens and Stephens’ expertise networks

Stephens and Stephens introduced the concept of “the expert network” laid upon the sociology of experience and expertise (SEE) framework to describe an interdisciplinary consortium of biologists, engineers, and physicists (Stephens and Stephens, 2021). Although this study focused on five postdoctoral associates, Stephens et al., use the SEE framework to distinguish between individuals with contributory expertise [ability to *do* the work] relative to interactional expertise [ability to communicate about the work, but *not directly do* it]. Graduate students arguably could take on either or both of these roles (Collins and Evans, 2007).

The preceding sketches indicate how these frameworks can provide a foundation to further examine the ways that graduate student engage in breakthrough science with non-student team members. However, these specifications need to be validated. We argue that key insights will come from examining science teams and team roles from a network perspective.

### Knowledge networks

A critical step toward assessing students’ role as boundary spanners is to examine the knowledge networks that graduate students actively join, construct, and navigate. In network literature, some networks and their characteristics have been described as knowledge networks, and more specifically as knowledge creation networks (Phelps et al., 2012). Knowledge creation is “the generation of new knowledge, typically in the form of ideas, practices, research papers, technical inventions, or products” (Phelps et al., 2012, p. 119). In the science of team science literature, the unique goal of knowledge creation distinguishes scientific and research teams from many other types of teams (Fiore, 2008). Knowledge networks do not happen by chance. Instead, they emerge through intentional cultivation of relationships among team members, including graduate students. Here, we highlight two strategies from peer-reviewed literature that team members may use to integrate graduate students into knowledge networks: network weavers and Archintors™. Graduate students themselves may also serve in these roles.

#### Network weaver

Holley (2012) provided a practical framework for “network weaving” that emphasized the intentional creation of connections that strengthen collaboration and knowledge exchange (Holley, 2012). Holley’s handbook positions network weaving as both a strategy and a mindset for cultivating resilient, inclusive networks that can drive innovation and social change. Essentially, a Network Weaver is someone who observes existing networks and facilitates their health from within or as an external catalyst. The emphasis is on empowering individuals and groups to self-organize, fostering trust, and enabling organic growth. Although the weaver has influence, the ultimate shape and direction of the network emerges from the collective interactions. “Weaving” implies a careful, intentional, and often iterative process of connecting existing threads to create a stronger, more beautiful, and functional fabric. It acknowledges the inherent complexity and organic nature of human relationships.

#### Archintor™

Scientific teams can be shaped by intentional design choices that influence interaction patterns, communication norms, and knowledge flow. Expectations, rules of engagement, and social positioning within teams all shape who participates, how knowledge is generated, and whose contributions are recognized. These dynamics are particularly important in the context of training and integrating graduate students into scientific discovery teams. To address structural exclusions and better integrate students into research networks, Love et al. (2023), introduced the concept of the Archintor™ (Love et al., 2023). An Archintor™ is a hybrid role that blends architectural design of team structures, instructional awareness, and facilitative leadership. As *architects*, Archintors™ design network configurations that reflect inclusive values and desired outcomes. As *instructors*, they guide the development of team capacity; as *facilitators*, they foster conditions for meaningful participation, especially for emerging scholars such as graduate students. In this model, the Archintor™ actively co-creates team expectations and ensures that students are not relegated to the periphery of research activity but are instead embedded as vital contributors in a robust knowledge network.

These strategies highlight that within science teams, research and education are inextricable. Collaboration simultaneously propels scientific discovery and nurtures emerging scientists. Fundamentally, research *is* education—it is the process by which we educate ourselves about our world and our place within it. Research is also a critical element in the education of students at all stages. Experiential learning, including research, has been shown to be a critical mechanism to help students find their passion (Kong, 2021; Thiry et al., 2011). Because graduate students occupy dual roles as learners and contributors, they are uniquely positioned to strengthen research teams’ knowledge networks and to shape their own professional trajectories through teaming.

## Preliminary field cases as a foundation for future research

The scenario provided in the Introduction is just one example that illustrates common challenges regarding graduate students’ roles on scientific teams. To advance needed research, we present a series of powerful vignettes followed by two field cases supported by social network data. Together, these observations indicate the unexplored variety and complexities of graduate student contributions to collaborative scientific discovery.

### Vignettes

New graduate students, especially those with no prior research experience, may begin their graduate careers without a clear concept of what research actually is—an educational process through the discovery of new knowledge. Consider this scenario: A new graduate student walks into a PI’s office and says, “I just collected the data you told me to collect. What do they mean?” Responding as a mentor, the PI might answer, “I don’t know. No one has ever done this experiment before, but let’s see if we can figure it out together.” This can spark an “ah ha” moment when the student realizes that their new data, collected in a novel experiment, is, by definition, the best data of its type *in the world.* Consider next the same student who, at some point later in their graduate career, walks into a PI’s office and reports, “I had an idea to redesign this experiment to see what would happen. Here’re the data I’ve collected, and I hypothesize that this means the following…” How might team-based research experiences have helped facilitate this transition? When thinking about the roles of graduate students on teams, a new graduate student may appear as the Maven, whereas through more experience they may move to a Salesperson role. Using Belbin’s model, the graduate student is acting as a Thinker and also Taking Action. Using Stephens and Stephens’ SEE/expertise network, the graduate student moves from interactional expertise to contributory expertise, not only through interactions with the PI but also, ideally, through teaming with others.

Another interesting observation about the graduate student learner-contributor is that their avenues for gathering information are not often apparent to more senior members of an interdisciplinary team. For example, during an interview, one of us asked a graduate student where they would get additional information for their interdisciplinary science team. The graduate student said she would go to her boyfriend’s roommate’s lab mate. Such weak ties, crucial for innovation (Granovetter, 1973), are often unrecognized and invisible to senior scientists. Indeed, these invisible ties are often ignored across academia but are apparent in Gladwell’s model as the Connector role and in Belbin’s model as the people social-oriented role cluster. Network weavers and Archintors™ specialize in structuring these less visible but powerful relationships.

Another vignette illustrates transdisciplinary innovation through network-based social learning where the graduate students fulfilled every role in multiple models (Aritzeta et al., 2007; Gladwell, 2000; Stephens and Stephens, 2021). In 2014, a chemist joined a transdisciplinary team with engineers, physicists, textile specialists, and clinicians. As is common in such collaborative efforts, the team focused on establishing a shared language and understanding. At one point, lab tours were proposed as a way of developing even deeper connections; graduate students were encouraged not only to participate but also to lead these tours. This opportunity sparked the curiosity of one chemistry graduate student, which led them to modify a commercial instrument in a clinical laboratory (Hawker et al., 2016). This was not something that the chemist or the clinician (or the engineer who helped the student on the design) had originally proposed, nor was it a critical component of the graduate student’s primary dissertation project. This kind of discovery and initiative represents the type of transdisciplinary integration needed to connect fundamental biomaterials research and clinical applications to solve wicked problems (Laursen et al., 2025).

Next, we illustrate the kinds of analyses that could deepen our understanding of how graduate students catalyze team networks. We present two cases with social network diagrams that illustrate the impact of integrating students and, by implication, the cost of their exclusion.

### Field case 1: the first connection: the emerging Archintor™

This case describes a graduate student’s role as an Archintor™ and Connector. As Gladwell (2000, p. 38–39) describes, “Connectors are people specialists. The strength of Connectors is that they know and maintain contact with many people. Connectors are people who link us to the world. People with a special gift for bringing the world together.” We suggest this student plays the Archintor™ as described above, although the intent of the student is not known.

In 2002, PI-1 was an assistant professor at a university in the Midwest of the United States. He was contacted by an energetic and excited young graduate student from another university [pseudonym: Theo Lane]. Theo asked for advice on some field techniques, and during the next year, they exchanged an occasional email. Theo had a unique idea about how viruses transferred between feline species and connected PI-1 with PI-2, a faculty member at a university in the Mountain West. She loved Theo’s idea, but PI-1, Theo, and PI-2 were at three different universities and a collaboration was too challenging to get off the ground.

In 2003, PI-1 moved to a university in the Mountain West for their dream job. Shortly after their arrival, Theo knocked on PI-1’s office door and announced that he had recently started at the university. Their collaborative research on feline viruses was now possible. In 2005, they decided to respond to a relevant program solicitation from the US National Science Foundation (NSF). Theo helped write the proposal with PI-1.

They submitted their proposal and received good reviews but were not funded in that round. Nevertheless, they continued to build their scientific team and resubmitted a revised proposal in 2006, only to be denied funding again. In 2007, they resubmitted their revised proposal and were finally funded. The team continued to grow, and new members joined as their research questions and plans evolved. In 2012, the team applied for and received a second, larger NSF grant to continue their research. In 2015, the university implemented a new initiative to study scientific teams, and their team became part of this new campus project. A science of team science (SciTS) scientist attended their team retreats and documented the team’s history and development from inception in 2002 to 2019. All data collection methods were performed with the informed consent of the participants and followed Institutional Review Board protocol #19-8622H at Colorado State University.

**FIGURE 1:**
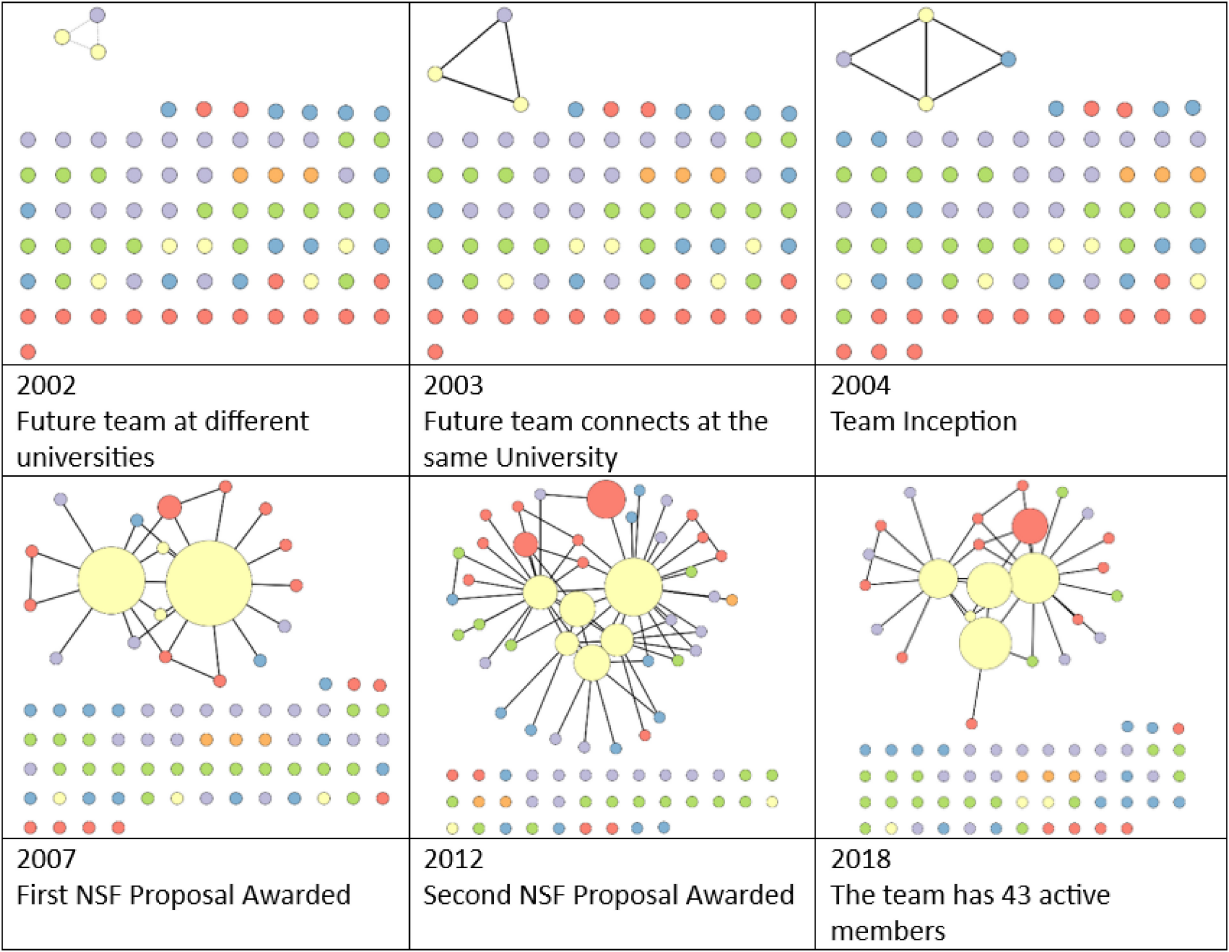
Team evolution milestones: The connected network component represents the active team members at each time point. The isolates (nodes not connected to the network) include every team member who participated from 2002 to 2019. Different colors represent different position titles (e.g., postdoc, PI).

Multiple social network diagrams illustrate the teams’ evolution from 2002 to 2018 (Figure 1).

It was not just PIs who were impacted by Theo acting as Connector, Catalyst, and Archintor™; graduate students and postdocs were also affected. In total, the team had 81 members over the time period studied. By 2019, they had 33 extramural awards totaling over 5.6 M USD and 58 peer-reviewed publications with coauthors from 39 different universities, 13 state agencies, and 11 other organizations. The team also trained 21 graduate students and 15 postdocs. Furthermore, the team was described as an exemplary case study in an article published in 2021, highlighting their development and accomplishments and how interpersonal connections have been a driver for their success (Love et al., 2021). This team exemplified high levels of success and transdisciplinary collaboration. It all began with a graduate student who connected two faculty members. Read the entire study in *Humanities and Social Sciences Communications* (Love et al., 2021).

### Field case #2: bridging disciplinary silos

This case describes how students bridged disciplines through coauthoring. Analysis of the data indicates that the graduate students in this team fulfilled Thinking and Action/Task-Oriented roles as they bridged disciplinary silos (Aritzeta et al., 2007). All data collection methods were performed with the informed consent of the participants and followed Institutional Review Board protocol #19-8622H at Colorado State University.

**FIGURE 2:**
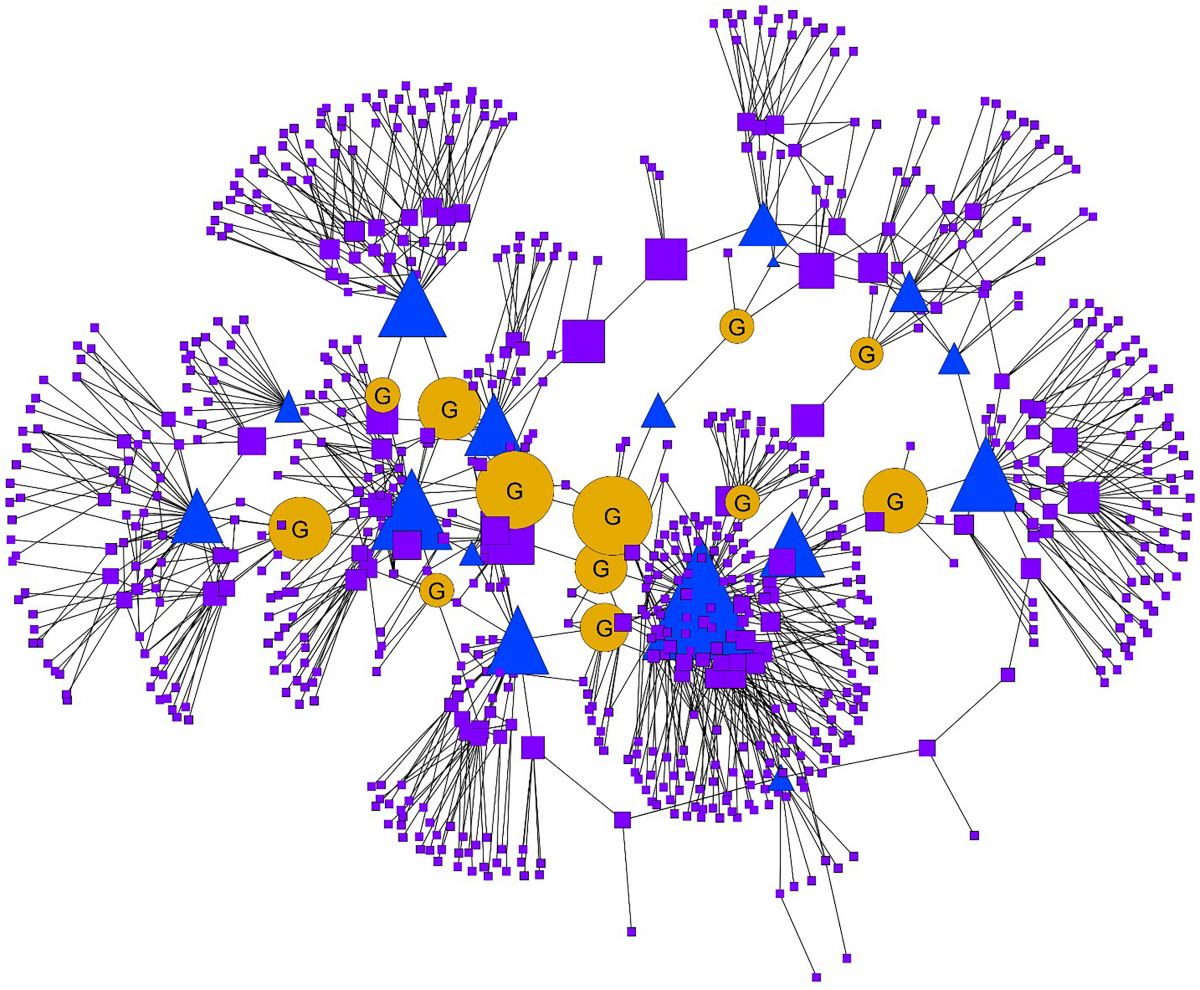
Graduate students are connectors: Gold circles with a “G” are graduate students with a betweenness score in the top 25. Faculty/PIs are blue triangles. All remaining nodes, postdocs, research associates, etc., are purple squares. The size indicates betweenness centrality where larger nodes have higher betweenness scores and smaller nodes have smaller betweenness scores.

We analyzed the co-authorship publication network of an interdisciplinary research team over a 2-year period (2010–2012) to examine patterns of collaboration. Figure 2 shows the resulting network distinguishing the 12 PIs on the interdisciplinary team (representing five colleges and six different departments) from their coauthors, some of whom were students and other trainees.

A primary goal with this analysis was to identify individuals who served as key brokers within the network structure. To achieve this, we examined betweenness centrality, a network metric that identifies individuals who serve as key intermediaries in the flow of information. The network shown in Figure 2 is relatively open with many nodes acting as bridges between different, more disconnected clusters. However, what is noteworthy is that 10 of the top 25 betweenness scores were held by graduate students. In fact, when we remove the 12 PIs from the top 25, 10 out of 13 of the remaining key boundary spanners were students—not postdocs, research staff, or external collaborators.

This finding highlights the pivotal role graduate students can play in facilitating scholarly collaboration and knowledge exchange across different subgroups within a network. Their positioning suggests that graduate students not only contribute to research output (contributory expertise) but also build bridges across disciplinary silos (interactional expertise) (Collins and Evans, 2007). They are essential Connectors who enhance the overall cohesion and information flow of the academic community. Cohesive networks encourage strong relationships thereby fostering collaboration and innovation as ideas are shared more freely (Phelps et al., 2012). Importantly, effective information flow ensures knowledge is disseminated throughout the network. This type of sharing is vital for learning and skill development as well as for the co-creation of new knowledge (Phelps et al., 2012; Singh, 2005).

The boundary-spanning experience of the trainees on this team has set them up to continue serving as key intermediaries through their research programs. Anecdotally, one of the graduate students with a high betweenness centrality score continues to foster interdisciplinary projects in their current role as a university research faculty member. A recent Web of Science search indicates this individual’s publications span such diverse research areas as chemistry, materials science, biology, radiology, nuclear medicine, medical engineering, energy/fuels, and art.

## Discussion and roadmap for future research

The vignettes and cases presented here represent only a few examples of how graduate students have catalyzed discovery, fulfilled a multitude of roles on teams, and established themselves as scientists through team knowledge networks. Too often, their efforts were unrecognized, and their contributions remained invisible, undervalued, and overlooked. Graduate students are frequently engaged in a range of team tasks including connecting disciplines, performing intellectual (and manual) labor, managing data infrastructures, and maintaining continuity across shifting research priorities. We hypothesize that they accomplish these tasks by fulfilling various boundary-spanning team roles such as Connector, Coordinator, and Interactional expert. Their work as network weavers and Archintors™ (intentional or unintentional) is likely critical for the health and productivity of scientific teams (Holley, 2012; Love et al., 2023). These field cases and vignettes show that additional research is needed.

A roadmap of future research opportunities involves multiple stages, and it starts with identifying teams that are inclusive of graduate students and comparing their outcomes with teams that are less inclusive. We recommend starting with these high level research questions: How do graduate students’ perceived and actual team roles relate to team performance outcomes? and What barriers limit the teams’ ability to leverage graduate student contributions?

The next stage involves conducting comparison studies to examine differences between teams that include graduate students and those that do not. High level research questions for these studies include: How does the underutilization of graduate students’ skills and team roles affect overall team performance? How does full inclusion of graduate students on teams support students becoming experts in their field or integration experts? (Hoffmann et al., 2024; Hoffmann et al., 2022).

As the final stage, research is needed to determine the roles that graduate students are playing on teams and to further understand the knowledge networks graduate students are supporting and creating. This research needs to include the examination of how these networks generate successful outcomes and breakthrough science. Based on the field cases in this article, studies grounded in a network perspective need to include longitudinal social network analysis and longitudinal outcome metrics of both the graduate student and team members. Recommended high level research questions include: What are the ideal network configurations for graduate students to be effective on a team? How do networks vary based on the role of graduate students on a team? At which timepoints is it critical to have particular roles filled by graduate students?

## Conclusion

We believe graduate students are not merely apprentices; they are emerging scientists, knowledge brokers, network weavers, and Archintors™. They are catalysts. Excluding them as recognized contributors on scientific teams represents a missed opportunity for innovation, equity, and learning. By analogy, community-based research describes the co-creation of knowledge wherein community partners are equitable contributors in the research. Imagine the impact if, like community members, graduate students were treated as co-creators of knowledge on scientific teams (Strand et al., 2003).

This article calls for future research and action addressing the untapped potential of graduate students as team catalysts. The next steps require sustained commitment, empirical testing, and systemic transformation to ensure that graduate students do not remain on the periphery of scientific discovery. Indeed, we hypothesize they are essential to breakthrough science.

## Data Availability

The datasets presented in this study can be found in online repositories. The names of the repository/repositories and accession number(s) can be found at: Figure 1 data: https://doi.org/10.25675/10217/214187.
